# AT1R blocker prevents mental stress induced retrograde blood flow in overweight/obese men

**DOI:** 10.14814/phy2.15566

**Published:** 2023-01-12

**Authors:** Helena N. M. Rocha, Gabriel F. Teixeira, Gabriel M. S. Batista, Amanda S. Storch, Vinicius P. Garcia, Juliana Mentzinger, Erika A. C. Gomes, Monique O. Campos, Antonio C. L. Nóbrega, Natália G. Rocha

**Affiliations:** ^1^ Department of Physiology and Pharmacology, Laboratory of Exercise Sciences Fluminense Federal University Niteroi Brazil; ^2^ Department of Physiology and Pharmacology, Laboratory of Integrative Cardiometabology Fluminense Federal University Niteroi Brazil; ^3^ National Institute of Science and Technology (INCT) ‐ Physical (In)activity and Exercise, National Council for Scientific and Technological Development (CNPq), Fluminense Federal University Niteroi Brazil

**Keywords:** blood flow, endothelin‐1, mental stress, nitric oxide, obesity, shear rate

## Abstract

The main goal was to determine the impact of mental stress (MS) on blood flow regulation in overweight/obese men. Fourteen overweight/obese men (27 ± 7 years; 29.8 ± 2.6 kg/m^2^) participated in two randomized experimental sessions with oral administration of the AT1R blocker Olmesartan (40 mg; AT1RB) or placebo (PL). After 2 h, a 5‐min acute MS session (Stroop Color Word Test) was administered. Blood flow was assessed at baseline and during the first 3 min of MS by vascular ultrasound in the brachial artery. Blood was collected before (baseline) and during mental stress (MS) for measurement of nitrite (chemiluminescence) and endothelin‐1 (ELISA kit). The AT1R blocker was able to reverse the MS responses observed in the placebo session for retrograde flow (*p* < 0.01), retrograde SR (*p* < 0.01) and oscillatory shear index (*p* = 0.01). Regarding vasoactive substances, no differences were observed in ET‐1 (*p* > 0.05) responses to MS between experimental sessions. However, for nitrite responses, the administration of the AT1R blocker was able to increase circulating levels of NO (*p* = 0.03) Blockade of AT1R appears to prevent the decrease in endothelial function by reducing low shear stress and maintaining the vasoactive substances balance after MS in overweight/obese men.

## 1 INTRODUCTION

Both obesity and mental stress (MS) are considered independent cardiovascular risk factors, underlying the development and progress of endothelial dysfunction and atherosclerosis (Bjorntorp, 2001). Obesity prevalence has increased exponentially, as research shows that by 2030 approximately 1 billion people around the world will be diagnosed with obesity (Organization WH, 2022). It was demonstrated that obese individuals subjected to chronic stress or major depression present increased susceptibility to worse cardiovascular outcomes (Chaddha et al., 2016).

During fight or flight response, the sympathetic nervous system plays a crucial role in increasing cardiac output and blood pressure. It also contributes to blood flow redistribution, prioritizing skeletal muscle areas, whereas reducing the blood flow in splanchnic and kidney areas (Loures et al., 2002). However, previous studies have demonstrated that blunted adrenergic stimuli did not restore the impaired endothelial and vascular response to stress (Halliwill et al., 1997). This evidence suggests that there are other key mediators, which regulate endothelial function and vascular tone during stressful situations. Renin‐angiotensin system represents a potential underlying mechanism, because it modulates several endothelial actions and seem to be overactivated during stress in healthy (Gideon et al., 2022) and obese individuals (Cooper et al., 1998; de Kloet et al., 2014). Angiotensin II (Ang II) and its main receptor [angiotensin II type 1 receptor (AT1R)] induces vasoconstriction and redox imbalance, contributing to endothelial dysfunction in the long‐term.

The endothelium is an endocrine organ capable of synthesizing many important vasodilators and vasoconstrictor substances, two key ones called nitric oxide (NO) and endothelin‐1 (ET‐1) responsible for vasodilation and vasoconstriction, respectively. The balance of these substances is crucial for the regulation of vascular tone (Furchgott & Zawadzki, 1980) and blood flow patterns (Favero et al., 2014). Laminar antegrade blood flow seems to mechanically stimulate the production of NO by endothelial nitric oxide synthase (eNOS) in a phenomenon known as shear stress (Padilla et al., 2008). While oscillatory or retrograde blood flow (RBF) leads to low shear stress, ergo less stimulation for NO production and increased susceptibility for development of atherosclerosis (Rocha et al., 2018). Moreover, RBF generates in imbalance in redox homeostasis by increasing reactive oxygen species (ROS) production, via enhanced gene expression and post‐transcriptional activity of nicotinamide adenine dinucleotide phosphate (NADPH), and downregulating intracellular scavengers such as superoxide dismutase and glutathione (Chatzizisis et al., 2007).

In vivo and in vitro experimental studies have demonstrated that increased concentrations of Ang II, as observed in response to MS (Saavedra et al., 2005), is related to the eNOS downregulation and therefore, decreased NO bioavailability (Le Brocq et al., 2008). Also, NO is a potent endogenous inhibitor of ET‐1 by modulating its transcription (Le Brocq et al., 2008). Additionally, in vitro studies showed that exposure to RBF provoked an increased AT1R expression at 12 and 24 h (Ramkhelawon et al., 2009).

Considering the deleterious effects of RBF on the endothelial cells and the association between stress and obesity seem to double the cardiovascular impairment (Pi‐Sunyer, 2009; Simon et al., 2011; van der Valk et al., 2018), it is imperative to understand the mechanisms and consequences of acute exposure to MS on blood flow regulation. We hypothesized that overweight/obese individuals under stress would present increased RBF along with imbalanced vasoactive substances, such as NO and ET‐1, and that the activation of the Ang II‐AT1R may play an important role in this phenomenon.

## 2 METHODS

### 2.1 Study population

After eligibility screening i.e., clinical history assessment, anthropometric and arterial pressure measurements and biochemical blood analysis interpretation, fourteen overweight/obesity grade I men (27 ± 7 years; 25 and 35 kg/m^−2^; body fat mass > 25%; Chaddha et al., 2016) were enrolled in the study. Inclusion criteria consisted of (1) the absence of any diagnosed disease, (2) no current pharmacological treatment, (3) a non‐smoker status/history and (4) a physically inactive lifestyle (<150 min per week of moderate‐intensity cardiorespiratory exercise training; Ministério da Saúde. DATASUS, 2011). The measurement of body composition by bioelectrical impedance analysis was performed using RJL Systems Body Composition software (Clinton Township). This study protocol was approved by the ethics committee of Fluminense Federal University (CAAE 76594217.0.0000.5243) and conformed to the standards set by the latest revision of the Declaration of Helsinki (except for registration in a database). All subjects gave written informed consent before their participation in the study.

### 2.2 Protocol and experimental design

The protocol consisted of a randomized, crossover, blind, and placebo‐controlled study. The experimental sessions consisted of oral administration of angiotensin II type 1 receptor blocker (40 mg, AT1RB; Olmesartan, lot number: 60818, Pfizer) or oral administration of placebo (PL; starch pill). All participants were subjected to both medications. In the 24 h prior to both sessions, subjects were oriented to maintain a light diet and abstain from alcohol, caffeine, and intense exercise. All experimental sessions took place in the morning.

After the subjects arrived, blood pressure was measured using the oscillometric method (Omron Dalian co, HEM‐7113), an intravenous catheter was placed in the antecubital cavity for blood sampling and a blind oral administration of AT1RB or PL was performed. A 2‐h resting period (the time to maximum plasma concentration of olmesartan following a 40 mg oral administration) in supine position was respected before the 5‐min MS task. Venous blood samples were collected before and during MS. Blood flow measurements were conducted at baseline and during the mental stress task. In the present study, beat‐by‐beat blood pressure and heart rate were recorded via photoplethysmography (Finometer, Finapres Medical Systems).

### 2.3 Mental stress

Mental stress tasks have been largely used as a simulation of mental or psychological stress situations in a standardized and controlled environment (Ghiadoni et al., 2000; Poitras & Pyke, 2013). An adapted version of the Stroop Color Word Test was applied for 5 min, preceded by 2 min of baseline measurement and followed by 3 min of recovery. The task consists of a slideshow that changes every 2 s projected on the ceiling above the subject, along with auditory conflicts (voices pronouncing colors different from those projected) that were continuously inflicted via headphones. A subjective scale of perceivable stress ranging from zero to four, was assessed after each test (0 = non‐stressful, 1 = not very stressful, 2 = stressful, 3 = very stressful and 4 = extremely stressful).

### 2.4 Blood flow measurements

Blood flow measurements were performed at baseline and in the last 30s of the first 3 min of MS. Blood sampling for evaluation of vasoactive substances was performed in the last 2 min of the MS task. Brachial artery blood velocity and diameter were recorded using a high‐resolution Doppler ultrasound system (LogiQ P5, GE Medical Systems) attached to a video capture board with USB 2.0 (Dazzle Video Capture, EUA) connected to a laptop computer, which enabled the ultrasound video signals to be real‐time encoded and captured at a frequency of 30 Hz. The sampled volume was located at the center of the brachial artery and then adjusted to the full vessel width. Artery diameter was analyzed offline with an automated edge‐detection and wall‐tracking software (Vascular Research Tools 5, Medical Imaging Applications; Fernandes et al., 2014; Sales et al., 2014).

### 2.5 Nitrite concentration

Venous blood samples were collected into trace metal‐free tubes containing sodium heparin. After centrifugation, plasma was deproteinized with cold ethanol and injected into a purge vessel containing iodide and acetic acid at room temperature, which is able to convert nitrite to NO by the ozone‐chemiluminescence method with a Nitric Oxide Analyzer (model 280i, Sievers; Metzger et al., 2006). The plasma nitrite concentration was determined by interpolation of a nitrite standard curve.

### 2.6 Endothelin‐1

The measurement of endothelin‐1 was determined by utilizing an immunometric ELISA kit (Endothelin ELISA kit, Cayman, EUA) using plasma isolated from venous blood samples collected into EDTA tubes, following manufacturer's instructions.

### 2.7 Calculations and statistical analysis

Blood velocity was recorded and analyzed in the Doppler ultrasound system. Blood flow was calculated from the mean blood velocity (*V*_mean_) and vessel area, considering 60 as a constant (i.e., *V*_mean_ × Area × 60). Shear rate (SR), a proxy of shear stress, was calculated as four times the ratio between mean blood velocity and the artery diameter [i.e., 4 × (*V*_mean_/diameter)]. The oscillatory shear index (OSI), which allows quantification of shear oscillations degree and ranges from 0 (no oscillation) to 0.5 (high oscillation), was calculated according to following formula: |retrograde shear|/(antegrade shear + |retrograde shear|; Jenkins et al., 2013). Vascular conductance was calculated from mean blood flow and mean arterial pressure (ml/min·mmHg^−1^).

Considering the retrograde blood flow results as the main outcome and the alpha error to 0.05, the power of the statistical test for a sample size of 14 individuals was 0.8. The Shapiro–Wilk test and homoscedasticity were performed by the Levene's test to verify the normal distribution of the variables. A paired student's *t*‐test was conducted to compare the absolute magnitude of response to MS (Δ = during MS − baseline) between both sessions. Fisher's post‐hoc test was performed when significant differences between group, time, and/or interaction, were observed. Cohen's *d* was used to assess size effect. Data were expressed as the mean ± standard error of the mean (SEM). A probability <5% was considered statistically significant in two‐tailed analyses. The statistical package used was Statistica (version 10.0, StatSoft Inc. 2011).

## 3 RESULTS

Fourteen men were included in the study, 7 overweight and 7 obese (27 ± 7 years; 92.3 ± 10.2 kg; 29.8 ± 2.60 kg/m^2^; 32.3% ± 3.18%). Hemodynamic and flow parameters during baseline are shown in Table 1. No differences were observed in stress responses between sessions for systolic (ΔPL, 13 ± 2 mmHg vs. ΔAT1RB, 12 ± 2 mmHg; *p* > 0.05), diastolic (ΔPL, 12 ± 2 mmHg vs. ΔAT1RB, 11 ± 2 mmHg; *p* > 0.05), mean blood pressure (ΔPL, 13 ± 2 mmHg vs. ΔAT1RB, 13 ± 2 mmHg; *p* > 0.05) and heart rate (ΔPL, 13 ± 2 bpm vs. ΔAT1RB, 11 ± 2 bpm; *p* > 0.05). No differences were observed in any of the other variables presented as well. In addition, no differences were observed in the perceived stress level between experimental sessions.

**TABLE 1:** Brachial artery and vasoactive substances parameters at baseline in overweight/obesity individuals after oral administration of placebo and AT1R blocker.

Variables	Placebo	AT1R blocker
SBP (mmHg)	123 ± 2	120 ± 2
DBP (mmHg)	76 ± 2	76 ± 3
MBP (mmHg)	92 ± 2	81 ± 7
Heart rate (bpm)	62 ± 2	64 ± 2
Diameter (cm)	0.413 ± 0.01	0.398 ± 0.02
Blood velocity (cm·s^−1^)	5.94 ± 0.65	5.80 ± 0.96
Blood flow (ml·min^−1^)	191.95 ± 21.42	190.76 ± 36.22
Antegrade blood flow (ml·min^−1^)	226.73 ± 25.04	231.79 ± 35.88
Retrograde blood flow (ml·min^−1^)	34.63 ± 8.47	41.66 ± 7.89
Mean shear rate (s^−1^)	58.73 ± 7.46	56.72 ± 8.86
Antegrade shear rate (s^−1^)	68.43 ± 7.55	69.34 ± 8.49
Retrograde shear rate (s^−1^)	9.67 ± 2.41	12.95 ± 2.19
Oscillatory shear index	0.116 ± 0.02	0.169 ± 0.02
Conductance (ml·min^−1^/mmHg)	2.36 ± 0.25	2.31 ± 0.39
Nitrite (μM)	0.50 ± 0.04	0.47 ± 0.03
Endothelin‐1 (pg/ml)	7.06 ± 0.11	6.94 ± 0.13

*Note*: Values are expressed as mean ± SEM.

Abbreviations: AT1R, angiotensin II type 1 receptor; DBP, diastolic blood pressure; MBP, mean blood pressure; SBP, systolic blood pressure.

**FIGURE 1:**
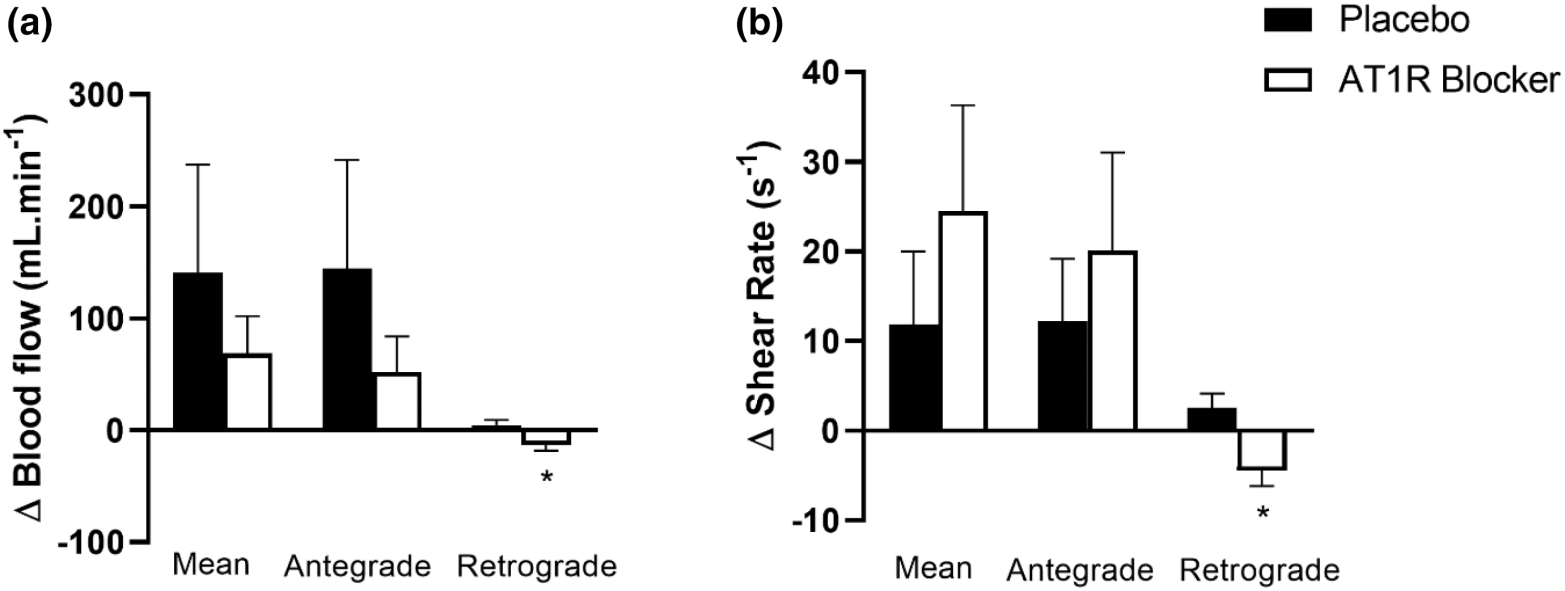
Mean, antegrade and retrograde blood flow (a) and shear rate (b) responses to mental stress [absolute magnitude of response to MS (Δ = peak blood flow during MS − baseline)]. AT1RB, angiotensin I type II receptor blocker. (*) *p* < 0.05 versus placebo.

The blood flow parameters are represented in Figure 1. No differences were observed in MS responses for mean (ΔPL, 140.50 ± 96.57 ml·min^−1^ vs. ΔAT1RB, 68.62 ± 33.29 ml·min^−1^; *p* = 0.35) and antegrade blood flow (ΔPL, 144.23 ± 97.25 ml·min^−1^ vs. ΔAT1RB, 52.17 ± 31.42 ml·min^−1^; *p* = 0.82). However, a decrease in the retrograde flow response (ΔPL, 4.55 ± 4.64 ml·min^−1^ vs. ΔAT1RB, −13.32 ± 4.84 ml·min^−1^; *p* < 0.01; Cohen's *d* = 1.08) was observed in the session with the AT1R blocker to MS. Similar to blood flow results, there was no difference in mean SR (ΔPL, 11.84 ± 8.14 s^−1^ vs. Δ AT1RB, 24.49 ± 11.82 s^−1^; *p* = 0.14) and antegrade SR (ΔPL, 12.19 ± 6.97 s^−1^ vs. ΔAT1RB, 20.08 ± 10.95 s^−1^; *p* = 0.31), however, during the session with AT1R blocker, the retrograde SR presented a decreased response to MS (ΔPL, 2.53 ± 1.58 s^−1^ vs. ΔAT1RB, −4.49 ± 1.70 s^−1^; *p* < 0.01; Cohen's *d* = 1.18).

**FIGURE 2:**
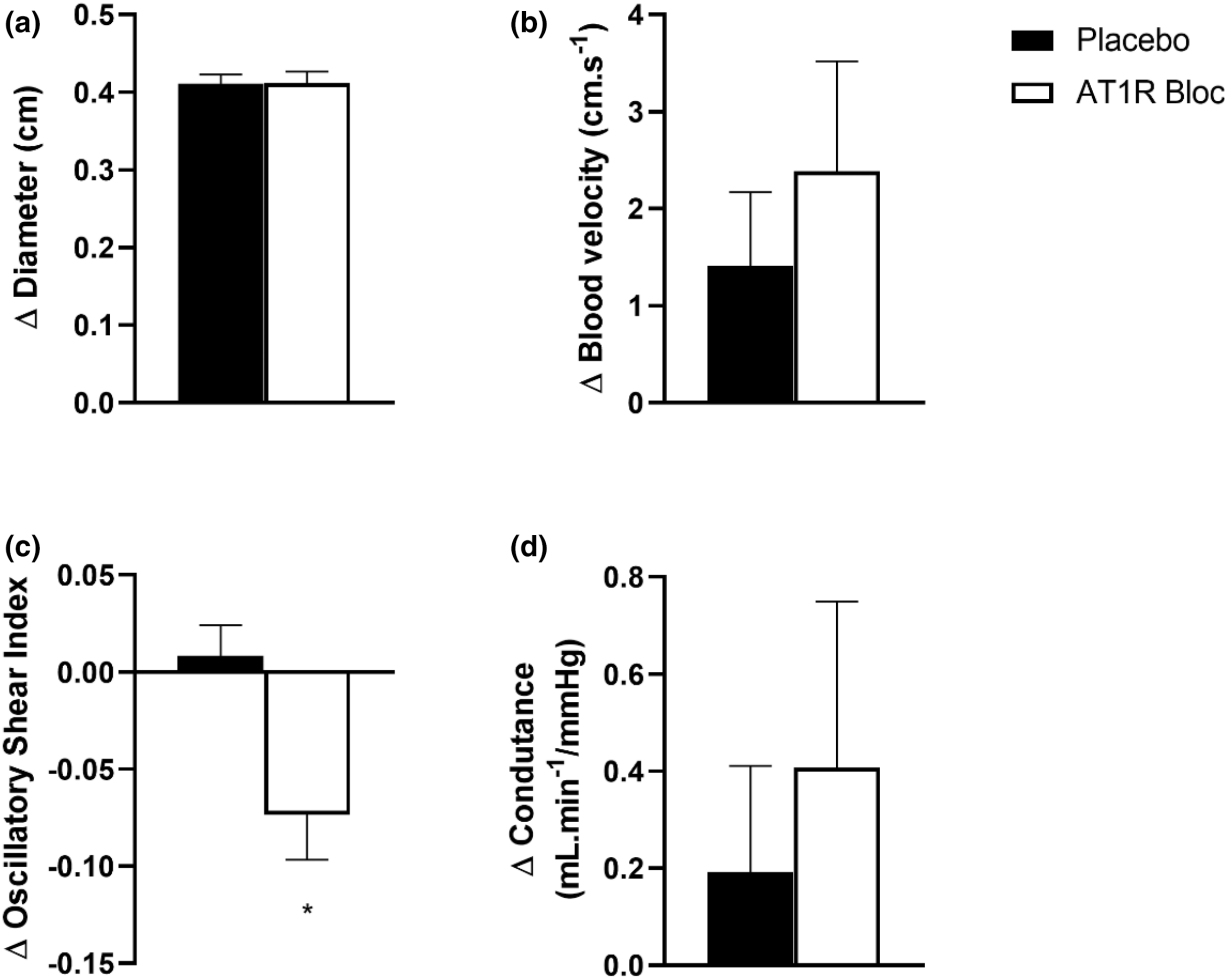
Diameter (a), blood velocity (b), oscillatory shear index (c) and vascular conductance (d) responses to mental stress [absolute magnitude of response to MS (Δ = peak blood flow during MS − baseline)]. AT1RB, angiotensin I type II receptor blocker. (*) *p* < 0.05 vs placebo.

Other blood flow parameters are represented in Figure 2. No differences were observed between the diameter responses (ΔPL, 0.07 ± 0.08 cm vs. ΔAT1RB, 0.04 ± 0.03 cm; *p* = 0.35), mean velocity (ΔPL, 1.41 ± 0.76 cm·s^−1^ vs. ΔAT1RB, 2.38 ± 1.13 cm·s^−1^; *p* = 0.25) and vascular conductance (ΔPL, 0.19 ± 0.21 ml·min^−1^/mmHg vs. ΔAT1RB, 0.40 ± 0.34 ml·min^−1^/mmHg; *p* < 0.01) to MS between sessions. On the other hand, we can observe that the session with AT1R blocker was able to decrease the responses of OSI (ΔPL, 0.09 ± 0.01 vs. ΔAT1RB, −0.07 ± 0.02; *p* = 0.01; Cohen's *d* = 0.82).

**FIGURE 3:**
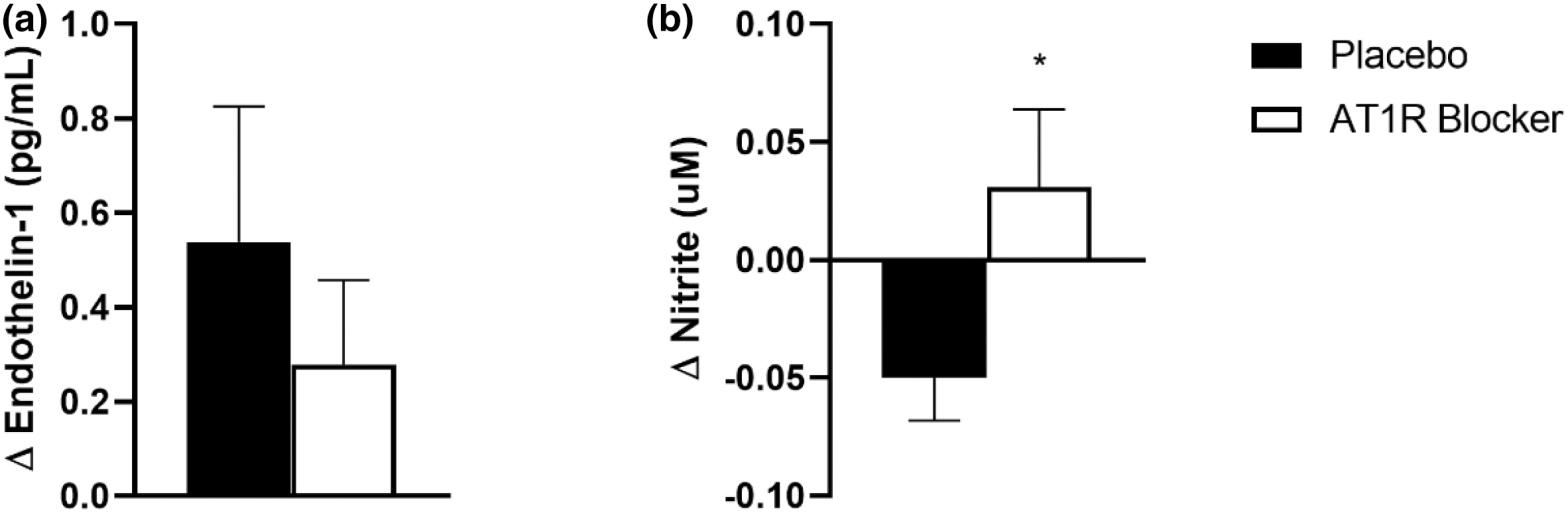
Endothelin‐1 (a) and nitrite (b) responses to mental stress [absolute magnitude of response to MS (Δ = peak blood flow during MS − baseline)]. AT1RB, angiotensin I type II receptor blocker. (*) *p* < 0.05 vs placebo.

Vasoactive substances are represented in Figure 3. No differences were observed in ET1 (ΔPL: 0.54 ± 0.29 pg/ml vs. ΔAT1RB: 0.28 ± 0.18 pg/ml; *p* > 0.05) responses to MS between experimental sessions. However, for nitrite responses, the administration of the AT1R blocker was able to increase circulating levels of NO (ΔPL: −0.05 ± 0.02 μM vs ΔAT1RB: 0.03 ± 0.03 μM; *p* = 0.03; Cohen's *d* = 0.90) to MS.

## 4 DISCUSSION

The present findings suggest that exposure to mental stress leads to decreased nitrite bioavailability, while the AT1R blocker leads to diminished retrograde blood flow and consequently low shear stress, in response to acute mental stress. No significant alterations were observed regarding mean velocity, diameter, mean and antegrade blood flow, and mean and antegrade shear. Several studies, including from our group, have used mental stress tasks as means to mimic stress situations in a controlled environment (Ghiadoni et al., 2000; Poitras & Pyke, 2013; Rocha et al., 2015; Sales et al., 2014; Spieker et al., 2002). The modified Stroop Color Word Test applied was able to inflict the same stressful stimulus in both sessions, as evidenced by similar increases in blood pressure and heart rate variables during the protocol.

Shear stress has a central role in vascular homeostasis because of its actions on the endothelial layer and, therefore, in the progress of atherosclerosis. In vivo studies have shown that areas exposed to RBF present greater risk of developing atherosclerotic plaques (Chatzizisis et al., 2007). RBF also augments cholesterol uptake and plaque instability (Andreou et al., 2015). Furthermore, in vitro studies provided extensive evidence regarding vascular remodeling and intracellular mechanotransduction (Chatzizisis et al., 2007). In the present study, contrary to what was observed with placebo, the AT1R blocker decreased the RBF in response to MS, which, in turn, decreased the SR, implying a potential protective effect of interruption of the AT1R pathway. As previously mentioned, the Ang II‐AT1R pathway activates the NADPH oxidase, increasing ROS and accumulation of superoxide anions, which is rapidly dismutaded to hydrogen peroxide, and leads to imbalanced redox homeostasis. It has also been shown that an increase in Ang II could inhibit eNOS expression and stimulate its uncoupling, which would, in turn, diminish NO bioavailability (Lin et al., 2014; Staiculescu et al., 2014).

In fact, exposure to MS provoked a decreased bioavailability of NO, which was not observed in the AT1R blocker session, whereas no changes were observed in ET‐1 response to MS. Nitric oxide is one of the main mediators of blood pressure response to MS (Trueb et al., 2012), due to the conjunction of impaired NO‐induced sympatho‐inhibition and loss of NO‐induced vasodilation. Studies show that forearm blood flow increases during MS and that this response is blunted by administration of L‐NMMA, an inhibitor of NO production by eNOS (Cardillo et al., 1997; Dietz et al., 1994). Oppositely, ET‐1 induces prolonged endothelial dysfunction in response to MS through ET_A_ activation (Spieker et al., 2002). Activation of AT1R has been reported to provoke pre‐proendothelin‐1 transcription, resulting in elevated ET‐1 levels (Lin et al., 2014). Moreover, Spieker et al. showed that the ET_A_ receptor blockade prevented flow‐mediated dilation impairment induced by mental stress (Spieker et al., 2002). Thus, when Ang II is released during MS, it is plausible to argue that both pathways are stimulated and act together leading to endothelial damage. It is important to highlight that inhibition of the sympathetic system does not improve endothelial function during or after MS (Halliwill et al., 1997) indicating that vasoactive substances such as NO and ET‐1 may be the main mediators of the endothelial response to MS. However, possibly due to a lack of statistical power, we did not observe alterations in ET‐1 concentrations.

The limitations of the present study relate mainly to the absence of women in order to avoid the established effects of sex hormones on the vascular function. Even though it is possible to minimize the hormone effect, the inclusion of women would deviate the study from its main goal, which was to understand a possible mechanism behind the endothelial dysfunction observed after mental stress. In addition, the lack of women in our subjects' group may be considered as a limitation concerning external validity of the results to the entire population, although it was an effective strategy to reduce confounding factors and thus enhance the internal validity of the study. Therefore, the present results do not allow us to infer that the same responses would be observed in women. Further, the same logic applies to extrapolation of these results for healthy individuals. The presence of a control/lean group could also enrich the study; however, the crossover, randomized and placebo‐controlled protocol is robust enough to attenuate this limitation. Moreover, considering that there is vast knowledge about the effects of stress on healthy subjects, we aimed to elucidate the mechanisms regarding subjects under cardiovascular risk. This is especially important because the association between stress and overweight/obesity duplicates the chances for a cardiovascular event (van der Valk et al., 2018). It should be mentioned that the AT1R blocker was not tested; however, literature shows that 40 mg of Olmesartan is likely to induce inhibition of AT1R (Miura et al., 2011; Stangier et al., 2001).

In conclusion, the present results indicate that MS influences blood flow regulation by increasing RBF and, therefore, LSS. It also leads to decreased NO bioavailability. Blocking the AT1R pathway blunted the effects induced by MS on blood flow and an opposite action on vasoactive substances. The present study provides new evidence about the role of Ang II‐AT1R in the deleterious response to mental stress and its consequence on blood flow regulation and endothelial health.

## AUTHOR CONTRIBUTIONS

All the experiments were performed in Laboratory of Exercise Sciences, located at Department of Physiology and Pharmacology, Biomedical Institute, Fluminense Federal University. The authors contributions were: (1) Conception or design of the work, Helena N. M. Rocha, Natália G. Rocha and Antonio C. L. Nóbrega; (2) Acquisition, analysis or interpretation of data for the work, Helena N. M. Rocha, Gabriel F. Teixeira, Gabriel M. S. Batista, Amanda S. Storch, Vinicius P. Garcia, Juliana Mentzinger, Erika A. C. Gomes, Monique O. Campos and Natália G. Rocha; (3) Drafting the work or revising it critically for important intellectual content: Helena N. M. Rocha, Gabriel F. Teixeira, Gabriel M. S. Batista, Amanda S. Storch, Vinicius P. Garcia, Juliana Mentzinger, Erika A. C. Gomes, Monique O. Campos, Antonio C. L. Nóbrega and Natália G. Rocha. It is important to mention that all listed authors are qualified for authorship; approved the final version of the manuscript; agreed to be accountable for all aspects of the work in ensuring that questions related to the accuracy or integrity of any part of the work are appropriately investigated and resolved.

## FUNDING INFORMATION

This work was supported by grants from the Brazilian National Council of Scientific and Technological Development (CNPq, 462,265/2014–5), the Foundation for Research Support of Rio de Janeiro (FAPERJ, E‐26/111.339/2014, E‐26/010.002170/2019, E‐26/202.770/2019) and Coordination for the Improvement of Higher Education Personnel (CAPES, scholarship).

## CONFLICT OF INTEREST

The authors report no conflicts of interest.
